# Determining the relationship between serum acute phase reactants and cervical premalignant lesions: a cohort study

**DOI:** 10.1590/1516-3180.2022.0186.29042022

**Published:** 2022-09-13

**Authors:** Yeliz Acar Sabır, Tayfur Çift, Süleyman Serkan Karaşin

**Affiliations:** I MD. Physician, Department of Obstetrics and Gynecology, University of Health Sciences Bursa Yüksek İhtisas Training and Research Hospital, Bursa, Turkey.; II MD. Physician, Department of Obstetrics and Gynecology, Health Sciences University Bursa Yüksek İhtisas Training and Research Hospital, Bursa, Turkey.; III MD. Physician, Department of Obstetrics and Gynecology, University of Health Sciences Bursa Yüksek İhtisas Training and Research Hospital, Bursa, Turkey.

**Keywords:** Squamous ıntraepithelial lesions, Uterine cervical neoplasms, Fibrinogen, Albumins, Procalcitonin, Cervical premalignant lesions, Low-grade squamous ıntraepithelial lesion, Cervical cancer screening

## Abstract

**BACKGROUND:**

Acute phase reactants play a role in the progression and prognosis of many malignant and premalignant tumors. This study investigated the diagnostic value of certain reactants as markers for cervical premalignant lesions.

**OBJECTIVES:**

Despite advanced screening and vaccination programs, cervical cancer remains a serious health problem worldwide. We aimed to determine the possible relationship between premalignant cervical disease and serum acute phase reactant levels.

**DESIGN AND SETTING:**

This study included 124 volunteers who underwent cervical cancer screening. We divided the patients into three groups according to cervical cytology and histopathological findings as follows: no cervical lesion, low-grade neoplasia, or high-grade neoplasia.

**METHODS:**

We included women aged 25–65 years with benign smear or colposcopy results, low- and high-grade squamous intraepithelial lesions. The benign group was based only on cytology findings, whereas the other groups were based on histopathology findings. Demographic data and serum albumin, fibrinogen, ferritin, and procalcitonin levels were evaluated in the three groups.

**RESULTS:**

We found significant differences among the three groups in terms of age, albumin level, albumin/fibrinogen ratio, and procalcitonin level. The regression analysis revealed lower serum albumin levels in the low- and high-grade squamous intraepithelial lesion groups compared with the benign group.

**CONCLUSION:**

This is the first study to evaluate the importance of serum inflammatory markers in cervical intraepithelial lesions. Our results indicate that serum albumin level, albumin/fibrinogen ratio, procalcitonin level, and neutrophil values differ among cervical intraepithelial lesions.

## INTRODUCTION

Cervical cancer is the third most diagnosed gynecological cancer and cause of death in the United States.^1^ Owing to advanced screening programs, cervical cancer currently has lower incidence and mortality rates compared with endometrial and ovarian cancer. However, cervical cancer remains a significant cause of cancer morbidity and mortality in countries with limited access to screening programs. Cervical cancer screening facilitates the detection and treatment of premalignant cervical lesions before cancer develops. Current screening methods include human papillomavirus (HPV) testing, cervical cytology (also known as the Pap test or Pap smear), or both.^2^

HPV is positively detected in 99.7% of patients diagnosed with cervical cancer.^2^ The infection of the cervical transformation zone with oncogenic HPV subtypes results in the initiation of carcinogenesis. The development of high-grade cervical intraepithelial neoplasia and invasive cancer from the first infection takes an average of 15 years, although rapid progression has also been reported.^3^

Inflammation reportedly plays an essential role in the formation, progression, and even invasion and metastasis of tumor cells. In reviewing the literature, we noted that numerous inflammatory complete blood parameters and acute phase reactants have been used in tumor diagnosis and prognosis.^4,5,6,7,8^

## OBJECTIVE

We aimed to evaluate the relationship between cervical premalignant lesions and serum albumin, fibrinogen, ferritin, and procalcitonin levels. These molecules play essential roles in systemic inflammation, as well as the detection of cervical premalignant lesions to a certain degree, considering the carcinogenesis process begins in the presence of HPV infection.

## METHODS

This case-control study included 124 volunteers with cervical cytology and histology results who applied to the Gynecology and Obstetrics department of the University of Health Sciences Bursa Yüksek İhtisas Training and Research Hospital between August 1, 2020, and March 19, 2021.

The University of Health Sciences Bursa Yüksek İhtisas Training and Research Hospital Clinical Research and Ethics Committee approved this study (approval number: 2011-KAEK-25 2020/07-04) on July 22, 2020. All participants read and signed the Informed Volunteer Consent Form.

We included women aged 25–65 years who applied to our gynecology department and had a benign smear or colposcopy result, low-grade squamous intraepithelial lesion (LSIL), or high-grade squamous intraepithelial lesion (HSIL). We categorized participants to the benign group only according to cytology findings and the other groups according to histopathology findings. We included patients who did not smoke or use alcohol or drugs; with no signs of active infection; and no known chronic, autoimmune, inflammatory diseases or cancer.

Patient age, gravidity, parity, polymerase chain reaction-based HPV DNA detection (HPV-DNA), and colposcopy results, if any, were recorded. Approximately 5 ml of blood was collected from each volunteer in four tubes. We determined and recorded the serum albumin, fibrinogen, ferritin, and procalcitonin levels in our patients using the appropriate kits in our hospital’s biochemistry laboratory. Results of pretreatment hemogram tests routinely performed upon admission were also obtained from the patient files. Leukocyte, hemoglobin, neutrophil, lymphocyte, mean platelet volume, and platelet levels were recorded. All final pathologies were evaluated by the Pathology department at our hospital. Conization pathology results of the treated patients were obtained and recorded. Patients with smear results showing benign findings (n = 53) were included in the control group.

Participants with low-grade lesions in the final pathology results were included in the LSIL group (n = 40); participants with high-grade lesions were included in the HSIL group (n = 28). Three patients with cervical cancer were excluded from the study. We compared the parameters recorded among the three groups using the appropriate statistical analyses.

### Sample calculation

Regarding the number of cases included in this study, we referred to a previous study titled “Diagnostic Value of Albumin to Fibrinogen Ratio in Cervical Cancer.”^9^ Based on the sample calculation using the fibrinogen and albumin parameters shown in Table 1, we included a total of 121 volunteers with a 95% confidence interval [CI] and 80% power. G*Power version 3.1.9.2 software (Erdfelder, Faul, & Buchner, 1996; Heinrich Heine University, Düsseldorf, Germany) was used for the sample calculation.

### Statistical analysis

SPSS version 24.0 for Windows (IBM Corp., Armonk, New York, United States) was used for the statistical analyses. Variables were examined visually (histograms, probability graphs) and using analytically (Shapiro-Wilk and Kolmogorov-Smirnov tests) to determine whether the data showed a normal distribution. Variables were defined as the mean ± standard deviation (X ± standard deviation), mean difference between groups, 95% confidence interval (95% CI), median (minimum-maximum), frequency (n), or percentage (%). Student’s t-test and the Mann-Whitney U test were used to compare normally and nonnormally distributed variables in the two-group analysis. Analysis of variance and Kruskal-Wallis tests were used to compare variables involving more than two groups. Pearson’s and Spearman’s tests were performed to determine the relationships between normally and nonnormally distributed variables. According to the cervical cytology and histopathology findings, independent predictors of benign, LSIL, and HSIL outcomes were analyzed using multinomial logistic regression analysis. The model compatibility was considered significant at P < 0.05. Receiver operating characteristic (ROC) curve analysis was performed to determine the borderline albumin value in patients who developed cervical intraepithelial lesions.

## RESULTS

The descriptive analysis results of the demographic and laboratory characteristics of the cases screened for cervical intraepithelial lesions are shown in Table 1. This study included 124 participants. The mean age of all participants was 40.1 ± 9.7 years. We conducted a further evaluation by biopsy in 18 patients with smear results indicating LSIL, 11 patients with HSIL, and 21 patients with atypical squamous cells of undetermined significance. The number of patients with benign cervical cytology findings was 68 (56.2%); 53 patients whose final pathology results were benign were included in the three-group analysis (42.7%). Despite benign smear findings, 15 patients underwent histopathological examinations because they were HPV-positive. Invasive cancer was detected in three patients, although these patients were not included in further analyses (Table 1).

**Table 1.** Comparison of demographic characteristics and laboratory parameters in benign and low- and high-grade squamous intraepithelial lesion groups according to cervical cytology and histopathology findings

	Benign (n = 53)	LSIL (n = 40)	HSIL (n = 28)			
	X± SD/Median (min-max)	X ± SD/Median (min-max)	X ± SD/Median (min-max)	P	P_Benign & LSIL_	P_Benign & HSIL_
Age (years)^*^	36.4 ± 9.7	41.2 ± 8.4	45 ± 9.2	< 0.01	0.043	< 0.01
Parity^#^	2 (0–9)	2 (0–9)	2 (0–9)	0.406		
Hemoglobin (g/dl)^#^	12.8 (9.9–15.1)	12.8 (7.9–15.6)	13 (7.4–15.4)	0.904		
Leukocyte count (mcl)^#^	7.7 (3.8–12.5)	7.1 (4.4–12)	7.5 (5.3–12.2)	0.536		
Neutrophil count (mcl)^#^	4.6 (1.6–9.3)	4.2 (2.1–11.8)	4.5 (2.8–8.2)	0.524		
Lymphocyte count (mcl)^*^	2.2 ± 0.5	2.2 ± 0.5	2.4 ± 0.8	0.462		
Mean platelet volume (fl)^*^	9.9 ± 0.9	9.9 ± 0.9	10 ± 1	0.834		
Platelet count (mcl)^#^	299 (113–453)	270 (164–437)	263 (171–498)	0.438		
Albumin (g/l)^*^	46.2 ± 2.9	44.6 ± 3.3	44 ± 3.4	**0.006**	**0.05**	**0.009**
Fibrinogen (mg/dl)^*^	309.8 ± 64.8	331 ± 85.7	331.6 ± 64.9	0.276		
Ferritin (ng/ml)^#^	29 (6–144)	26 (5–259)	26 (2–127)	0.895		
Procalcitonin (ng/ml)^#^	0.02 (0.01–0.06)	0.03 (0.02–0.32)	0.03(0.02–0.38)	**0.006**	**0.017**	0.149
Albumin/fibrinogen ratio^#^	14.5 (8.1–23.5)	14.3 (7.9–23.2)	13.6 (8.3–19.7)	**0.067**	0.295	**0.024**
Neutrophil/lymphocyte ratio^#^	2 (0.9–4.9)	2 (0.9–5.9)	2 (1–3.7)	0.905		
Platelet/lymphocyte ratio^#^	127 (43.4–206)	119.1 (64.6–238.1)	119.7 (68.5–233.1)	0.788		

Descriptive analyses were performed using the mean and standard deviation (X ± standard deviation) for normally distributeddata and median and minimum–maximum values for nonnormally distributed data. Statistical significance was set at P < 0.05. For two-group analyses of the results that were significant in the multiple regression analysis, Gabriel tests were used when variances from *post hoc* tests were homogeneously distributed, and Games-Howell tests when they were not. Homogeneity of variances was evaluated using the Levene's test.

^*^One-way analysis of variance

^#^Kruskal Wallis test.

HSIL = high-grade squamous intraepithelial lesion; LSIL = low-grade squamous intraepithelial lesion; min-max = minimum–maximum; SD = standard deviation. g/dl: gram/deciliter, mcl: microliter, fl: femtoliter, g/l: gram/liter, mg/dl: milligram/deciliter, ng/ml: nanogram/milliliter.

P values < 0.05 were considered significant.

We divided the included patients into benign, LSIL, and HSIL groups according to cervical cytology and final histopathology results. There was a statistically significant difference between the groups in terms of age, albumin level, procalcitonin level, and albumin/fibrinogen ratio (P < 0.01, P = 0.006, P = 0.006, and P = 0.067, respectively). When we evaluated the groups using binary *post hoc*, a statistically significant difference was observed between the benign and LSIL groups in terms of age, albumin, and procalcitonin values (P = 0.043, P = 0.05, P = 0.017, respectively). Binary *post hoc* analysis between the benign and HSIL groups showed statistical significance in terms of age, albumin level, and albumin/fibrinogen ratio. Meanwhile, procalcitonin levels did not differ between the two groups. No significant differences were observed in the laboratory parameters between the LSIL and HSIL groups (Table 1).

To determine the most compatible independent predictive variable for cervical intraepithelial lesions, multiple regression analysis was performed among the three groups, and the results are presented in Table 2. Although the benign cervical lesion group was the reference category, according to the multinomial logistic regression analysis, albumin values were significantly lower in the LSIL and HSIL groups than those in the reference group (P = 0.042 and P = 0.027, respectively). Each one-unit decrease in albumin level caused a 0.8-fold increase in the development of LSIL and HSIL (Table 2).

**Table 2.** Multinomial logistic regression analysis in benign and low- and high-grade squamous intraepithelial lesion groups according to cervical cytology and histopathology findings

Diagnosis	Parameters	B	Wald	OR	95% CI	P
Low-grade squamous intraepithelial lesion	Albumin (gr/l)	-0.165	4.144	0.848	0.705–0.957	0.042
	Albumin/Fibrinogen Ratio	-0.39	0.302	0.962	0.838–1.104	0.582
	Procalcitonin (ng/ml)	28.472	2.803			0.094
High-grade squamous intraepithelial lesion	Albumin (gr/l)	-0.198	4.921	0.820	0.689–0977	0.027
	Albumin/Fibrinogen Ratio	-0.79	0.967	0.924	0.789–1.082	0.325
	Procalcitonin (ng/ml)	27.413	2.580			0.108

CI = confidence interval; OR = estimated relative risk. Wald = test statistic value.

Multinomial logistic regression was used because the dependent variable consisted of three groups. The benign group was the reference category. Parameters that were found to be significant in the previous analysis were included in this analysis. The model fit was determined as P < 0.05.

g/l: gram/liter, mg/dl: milligram/deciliter, ng/ml: nanogram/milliliter.

P values < 0.05 were considered significant.

**Figure 1:**
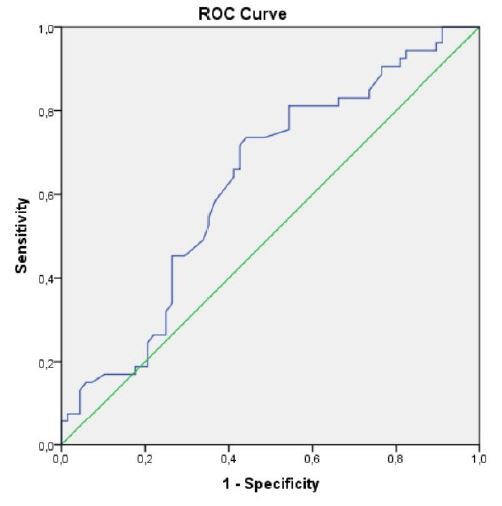
Receiver curve characteristic analysis of albumin values in patients with cervical intraepithelial lesions.

Receiver operating characteristic curves were created for the albumin parameter, and its predictive effect on the development of LSIL and HSIL was determined. The areas under the curve (AUCs), sensitivity, and specificity were calculated. The cutoff value for albumin was based on the values in the benign group. Accordingly, if the patient’s albumin level was < 46.05 g/L, a cervical intraepithelial lesion was expected with 65% probability, 65.9% sensitivity, and 60% specificity (AUC: 0.651 [0.553–0.750], P: 0.004) (Table 3, Figure 1).

**Table 3.** Receiver operating characteristic curve analysis result for albumin values in patients with cervical intraepithelial lesions following regression analysis results

Area under ROC curve (95% confidence interval)	Negative predictive value	Positive predictive value	Sensitivity	Specificity	Cutoff	P
0.651 (0.553–0.750)	60.1%	67.7%	65.9%	60%	46.05	0.004

ROC = receiver operating characteristic.

In addition, we divided HPV-DNA-positive patients (n = 68) into two groups: histopathologically-diagnosed LSIL (n = 40) and HSIL (n = 28). Accordingly, high neutrophil levels in women who are positive for HPV were associated with HSIL lesions (P = 0.041) (Table 4).

**Table 4.** Comparison of human papillomavirus-positive patients diagnosed with low- and high-grade squamous intraepithelial lesions according to laboratory parameters

	LSIL (n = 40)	HSIL (n = 28)	
	X ± SD/Median (min-max)	X ± SD/Median (min-max)	P
Hemoglobin (g/dl)^*^	13.4 (9.7–15.6)	13.1 (10.2–15.4)	0.891
Leukocyte count (mcl)^#^	7.1 (4.4–9.5)	7.5 (5.3–12.2)	0.075
Neutrophil count (mcl)^#^	4 (2.1–6.1)	4.5 (2.8–8.2)	0.041
Lymphocyte count (mcl)^*^	2.07 ± 0.6	2.5 ± 0.9	0.101
Mean platelet volume (fl)^*^	10 ± 1.05	10.1 ± 1.1	0.853
Platelet count (mcl)^#^	262 (191–331)	256 (181–498)	0.704
Albumin (gr/l) ^*^	45.1 ± 3.1	44.2 ± 3	0.362
Fibrinogen (mg/dl) ^*^	355.1 ± 76.6	330.2 ± 65.4	0.297
Ferritin (ng/ml) ^#^	30 (8–68)	29 (6–84)	0.574
Procalcitonin (ng/ml) ^#^	0.03 (0.02–0.32)	0.03 (0.02–0.38)	0.408
Albumin/fibrinogen ratio^#^	13.2 (8.2–19.2)	13.1 (8.3–19.7)	0.584
Neutrophil/lymphocyte ratio^#^	2 (0.9–3.7)	2 (1–3)	0.99
Platelet/lymphocyte ratio^#^	119.5 (70.3–238.1)	117.8 (68.5–172.8)	0.378

LSIL = low grade squamous intraepithelial lesion; HSIL= high grade squamous intraepithelial lesion; SD = standard deviation, min–max = minimum–maximum.

Descriptive analyses are presented using the mean ± standard deviation (X ± SD), median (minimum–maximum), and (n, %) for normally distributed, nonnormally distributed, and categorical variables, respectively.

^*^Student’s t-test

^#^Mann-Whitney U test

P < 0.05 were considered significant.

g/dl: gram/deciliter, mcl: microliter, fl: femtoliter, g/l: gram/liter, mg/dl: milligram/deciliter, ng/ml: nanogram/milliliter.

P values < 0.05 were considered significant.

In this study, 32 patients underwent conization for the final diagnosis. We divided these patients into LSIL (n = 13) and HSIL (n = 19) groups after pathological examinations. Comparison tests were performed in terms of laboratory parameters in these two groups. Accordingly, plasma leukocyte and neutrophil levels were statistically significantly higher in the HSIL group than those in the LSIL group (P < 0.05) (Table 5).

**Table 5.** Comparison of low- and high-grade squamous intraepithelial lesions according to laboratory parameters in patients who underwent conization

	LSIL (n = 13)	HSIL (n = 19)	
	X ± SD/Median (min-max)	X ± SD/Median (min-max)	P
Hemoglobin (g/dl)^#^	12.3 (9.7–15.6)	12.9 (10.2–15.4)	0.570
Leukocyte count (mcl)^#^	7.1 (4.4–10.6)	8 (5.3–12.2)	**0.030**
Neutrophil count (mcl)^#^	3.4 (2.1–6.9)	4.8 (2.8–8.2)	**0.014**
Lymphocyte count (mcl)^*^	2.1 ± 0.7	2.3 ± 0.8	0.497
Mean platelet volume (fl)^*^	10.2 ± 0.8	10 ± 1	0.509
Platelet count (mcl)^#^	262 (194–310)	259 (171–498)	0.910
Albumin (gr/l)^*^	45 ± 3	43.1 ± 3.5	0.126
Fibrinogen (mg/dl)^*^	362.7 ± 67.3	336 ± 60.4	0.249
Ferritin (ng/ml)^#^	35 (8–50)	31 (6–127)	0.762
Procalcitonin (ng/ml)^#^	0.03 (0.02–0.30)	0.03 (0.02–0.15)	0.880
Albumin/fibrinogen ratio^#^	12.8 (8.2–18.3)	12.7 (8.3–19.7)	0.791
Neutrophil/lymphocyte ratio^#^	2.1 (0.9–2.6)	2 (1–3.7)	0.323
Platelet/lymphocyte ratio^#^	118.1 (64.6–238.1)	118.9 (69.7–189.4)	0.910

LSIL = low grade squamous intraepithelial lesion; HSIL= high grade squamous intraepithelial lesion; SD = standard deviation; min–max = minimum-maximum.

Descriptive analyses are presented using the mean ± standard deviation (X ± SD), median (min–max), and (n, %) for normally distributed, nonnormally distributed, and categorical variables, respectively.

^*^Student’s t-test

^#^Mann-Whitney U test; P < 0.05 were considered significant.

g/dl: gram/deciliter, mcl: microliter, fl: femtoliter, g/l: gram/liter, mg/dl: milligram/deciliter, ng/ml: nanogram/milliliter.

P values < 0.05 were considered significant.

## DISCUSSION

This case-control study aimed to compare inflammatory markers between women with and without cervical intraepithelial lesions. Serum albumin levels were significantly lower in women with premalignant lesions compared with women with benign lesions.

Cervical intraepithelial neoplasms are premalignant squamous lesions of the cervix that are diagnosed by cervical biopsy and histological examination. These premalignant lesions of the cervix have undergone terminological changes over time and have since been revised. In the last classification system, the histological findings of the cervix are presented using the same terminology as the cytological findings.^10^

In women in developing countries, cervical cancer is the second most common type of cancer and the third most common cause of cancer-related death.^11^ Cervical intraepithelial lesions or invasive tumors are almost entirely caused by HPV infection. Cytological sampling of the cervix or HPV detection is the most effective screening method for cervical cancer. In particular, with the frequent use of HPV vaccines, screening programs have brought forth new perspectives for cervical cancer prevention.^12^

Early diagnosis after screening is vital for controlling the development of cervical cancer. However, an effective tumor marker for the early diagnosis, prognostic evaluation, and follow-up of patients with cervical cancer has not yet been established. The cytology and histological examinations mentioned above can sometimes show low sensitivity.^9^ Therefore, there is a need to develop cost-effective specific tumor markers for cervical lesions and early cervical cancer diagnosis. Furthermore, additional tests that can increase the sensitivity of various screening methods can improve their clinical applications.

We found some differences in laboratory values for whole blood markers and biochemical acute phase reactants in patients with cervical LSIL and HSIL compared with those of benign patient groups. Several factors are involved in the pathogenesis of cancer and pre-cancerous lesions. The relationship between cancer and inflammatory processes and the role of inflammation-related parameters in this pathogenesis have been demonstrated in many studies.^13,14,15^

A study by Sattar et al. determined that serum albumin levels in nonsmall cell lung cancer were lower than those in a healthy population.^16^ Moreover, Erlinger et al. reported that serum C-reactive protein levels were higher in patients with colorectal cancer compared with a healthy group.^17^ Although studies show that plasma fibrinogen elevation is significantly increased in many malignancies, previous studies and meta-analyses that evaluated the albumin and fibrinogen parameters together revealed that the proportional values of these two molecules differ significantly among cancer patients.^18,19,20,21,22 23^ In a 2016 study, plasma ferritin levels had a significant relationship with prostate cancer.^7^ A review published in 2016 that analyzed 15 articles found that serum procalcitonin levels are significantly valuable for the early diagnosis of infection-related complications and exacerbations in cancer patients.^24^ This finding indicates that although the procalcitonin molecule is not a direct cancer marker, it may differ significantly in malignancies compared with healthy populations and may be a topic of further study. However, previous studies have shown that serum procalcitonin levels increase significantly in lung, medullary thyroid, and metastatic liver cancer.^25,26,27^

Complete blood parameters have been extensively studied in many malignancies because they are easy to obtain, readily available, economical, and have pioneered many studies in the literature. Hemoglobin level, white blood cell count, and thrombocyte count and their relative values, which were among the complete blood parameters, also differed significantly in cancer patients compared with a healthy population.^6,28,29,30^ An article published in 2019 showed that serum neutrophil/lymphocyte and platelet/lymphocyte ratios were significantly associated with cervical cancer and cancer stage.^31^

Although studies and reviews have been conducted on the relationship between cancer, inflammation, and inflammatory molecules, the mechanisms that can assist in monitoring and screening cancer and precancerous lesions are unclear. We analyzed inflammation-associated whole blood parameters and other markers to observe cervical intraepithelial lesions exposed to HPV-related infection and inflammation processes.

In our study, serum albumin levels were lower and procalcitonin levels were higher in women who developed cervical intraepithelial lesions compared with a normal population. However, the plasma albumin/fibrinogen ratio was lower in the groups with cervical premalignant lesions than that in the healthy group. We performed a regression analysis to identify the data showing the most significant difference in cervical intraepithelial lesion development based on these three parameters. Serum albumin levels are the most significant laboratory finding with predictive value in women who develop LSIL and HSIL compared with a healthy population. With a sensitivity of 65% and specificity of 60%, the probability of developing cervical premalignant lesions increases in women with serum albumin levels below 46.05 g/l. Although studies have shown that serum albumin levels are low in some malignancies, this is the first analysis to detect significantly lower levels in cervical cancer precursor lesions. Universally accepted cervical smears and HPV tests are available for cervical cancer screening. As this is the first study of its kind, the differences in laboratory markers examined in our study may contribute to improved observation protocols after screening tests, disease prediction, and treatment evaluation, and may also serve as a reference for future studies on this topic.

A previous study reported that plasma albumin values differed significantly among patients with cervical cancer. In our study, we compared a population diagnosed with cervical intraepithelial lesions and a healthy population by observing the cytological and histological results for cervical cancer screening. In this sense, we are the first to describe the detection of low albumin values and albumin/fibrinogen ratios in women diagnosed with LSIL and HSIL in the literature. However, this analysis had some limitations. This study was conducted with volunteers at a single center, and the vaginitis or cervicitis findings of the patients could not be clearly evaluated. However, the number of patients diagnosed with cervical cancer was insufficient; therefore, this group was excluded from the study. A multicenter clinical prospective study with a larger cohort is needed to support the findings of this study.

Serum procalcitonin levels have been previously studied in solid tumors and significant results have been obtained. To our knowledge, our study is the first to correlate cervical lesions with procalcitonin levels. High procalcitonin levels were observed more often in women with LSIL compared with women with a healthy cervix. Previous studies have indicated that procalcitonin is a good indicator of infection in patients with cancer. However, it is not a validated diagnostic method, and the standard limit has not yet been defined. However, we believe that its use in daily clinical practice, preferably in combination with other clinical or laboratory tests, may help detect malignancies or premalignant lesions.

We divided the patients into two groups according to histopatho-logical examination results: LSIL and HSIL groups according to the histopathological examination results of participants who were HPV-DNA positive. We observed that the neutrophil count was higher in the HSIL group. In addition, when we evaluated the conization patients in the LSIL and HSIL groups, we found that the serum neutrophil count was higher in the HSIL group. This increase in cervical lesions and serum neutrophil values indicates that this parameter may be a supportive method for screening or observations. Previous studies have reported a relationship between serum neutrophil and neutrophil/lymphocyte ratio and inflammation. Increased neutrophil concentration is considered to promote neoplastic progression.^31,32,33^

This study investigated the relationship between inflammatory blood markers and precursor lesions in women screened for cervical cancer. Among these parameters, albumin/fibrinogen ratio and albumin, neutrophil, and procalcitonin levels were significantly associated with cervical intraepithelial lesions. However, these results may not directly indicate the role these molecules play in cervical cancer screening and may not indicate a direct relationship between the disease and its severity. This study was based on the observation of these laboratory parameters in a healthy population and a population with cervical lesions. Our findings may gain further significance by observing a larger group of patients, conducting additional multicenter studies, and incorporating different molecules into the analysis. To the best of our knowledge, this is the first such study in the literature.

## CONCLUSION

In conclusion, there is no low-cost, highly-specific diagnostic laboratory marker that can assist the smear and HPV tests used in screening for cervical cancer and intraepithelial lesion precursors to cervical cancer. Low serum albumin levels may be predictive of cervical lesion development. Future studies with more patients and different study designs may provide new data for the prediagnosis and follow-up of premalignant cervical diseases. This study is the first to evaluate the importance of serum inflammatory markers in cervical intraepithelial lesions. According to our results, serum albumin, albumin/fibrinogen ratio, procalcitonin, and neutrophil values were significantly correlated in cervical intraepithelial lesions.
